# Understanding the role of preservice teachers’ attachment in shaping core self-evaluations: the mediating effect of academic emotions in teacher education contexts

**DOI:** 10.3389/fpsyg.2026.1717612

**Published:** 2026-01-29

**Authors:** Yuanyuan Zhang, Shuhui Xu

**Affiliations:** Department of Psychology, Wenzhou University, Wenzhou, China

**Keywords:** academic emotions, attachment, core self-evaluations, preservice teachers, professional development, teacher education

## Abstract

**Background:**

Core self evaluations (CSE) are critical for preservice teachers’ professional functioning, yet the emotional and attachment related processes that shape CSE remain understudied. This study tested whether academic emotions mediate associations between attachment orientations and CSE.

**Methods:**

A sample of 307 preservice teachers completed self report measures of attachment anxiety and a composite of closeness and dependence, six discrete academic emotions (disappointment, interest, pride, shame, hope, pleasure), and the Core Self Evaluations Scale. We conducted correlational analyses, hierarchical multiple regression, and bootstrapped mediation analyses.

**Results:**

Attachment orientations, academic emotions, and CSE were significantly intercorrelated. In hierarchical regression, attachment anxiety, the closeness and dependence composite, and the six academic emotions together accounted for 47% of variance in CSE (R^2^ = 0.47). Disappointment emerged as the strongest negative predictor of CSE. Bootstrapped mediation models indicated that disappointment fully mediated the negative association between attachment anxiety and CSE, whereas interest and pride jointly mediated the positive association between the closeness and dependence composite and CSE.

**Conclusion:**

Academic emotions, particularly disappointment, play a central role in translating attachment orientations into preservice teachers’ core self evaluations. Interventions that reduce academic disappointment and cultivate interest and pride, alongside efforts to foster secure attachment related experiences, may bolster preservice teachers’ professional self evaluations.

## 1 Introduction

Core self-evaluations (CSE) denote individuals’ broad, foundational appraisals of their own competence and worth. In recent years CSE has attracted considerable scholarly interest because it reliably predicts job satisfaction, psychological wellbeing, job performance, and career commitment (Bono and Judge, 2003; Di Fabio and Palazzeschi, 2020). CSE comprises four core components, including self-esteem, locus of control, generalized self-efficacy, and emotional stability, which together reflect stable beliefs about one’s fundamental personal resources (Yang, 2015). Recent empirical and review studies have extended these links to educational contexts, reporting that teacher wellbeing, autonomy support, and workplace resources are closely associated with teachers’ CSE and with downstream classroom outcomes for students (Forster et al., 2022; Collie, 2023; Liang et al., 2020).

In educational contexts, CSE has been linked not only to teachers’ job satisfaction, emotional exhaustion, and burnout, but also to teaching efficacy, occupational resilience, and perceived social support (Sun et al., 2011; Yang et al., 2013; Zhao and Wang, 2016). Teachers’ CSE may also indirectly shape students’ motivation and classroom engagement through teacher–student interactions (Ma and Li, 2009; Xue et al., 2025). Importantly, recent randomized and quasi-experimental studies indicate that scalable interventions targeting educator wellbeing can produce sustained improvements in psychological distress and related competencies, which in turn have positive implications for teaching quality and student outcomes (Hirshberg et al., 2022; Dreer, 2023). These findings highlight CSE not only as a dispositional predictor but also as a malleable resource with direct relevance for teacher preparation and retention.

For preservice teachers, core self-evaluations are closely associated with academic performance, psychological wellbeing, and professional identity formation, with important implications for teaching quality and student outcomes. Growing evidence indicates that teacher wellbeing is positively related to self-efficacy, instructional quality, and student wellbeing, and that targeted interventions can sustainably enhance educators’ psychological resources (Dreer, 2023; Hirshberg et al., 2022). Nevertheless, extant research has concentrated disproportionately on in-service teachers, leaving open how foundational self-evaluative resources are formed during the preservice period and how early relational patterns influence these resources in culturally specific educational systems such as China’s teacher-training environment. This gap constrains the design of early, preventive, and culturally attuned supports for teacher candidates.

Accordingly, the present study examines attachment patterns and academic emotions, focusing on the mediating role of academic emotions in the association between attachment and core self-evaluations. Methodologically, the study addresses this question by modeling multiple discrete academic emotions and testing indirect effects using robust bootstrapped mediation procedures, thereby providing more nuanced evidence on which affective pathways transmit attachment influences into core self-evaluative beliefs. Substantively, the study contributes to the literature by situating these processes in a Chinese preservice teacher sample, clarifying cultural boundary conditions for theoretical models that link relational history, affective experience, and professional self-views.

## 2 Theoretical framework

### 2.1 Attachment and Core self-evaluations

Attachment theory holds that early interpersonal experiences with primary caregivers lead to the formation of internal working models—cognitive–affective representations of the self and others—that guide emotion regulation, self-concept, and interpersonal functioning across the lifespan (Bowlby, 1979; Eilert et al., 2023). In adulthood, individual differences in attachment are reliably conceptualized along dimensions of attachment anxiety and attachment avoidance, which systematically influence trust, comfort with closeness, and patterns of emotional regulation in close relationships, with secure attachment associated with more adaptive regulation and interpersonal functioning relative to anxious and avoidant orientations (Alfasi and Besser, 2024; Eilert et al., 2023).

Attachment anxiety is marked by relational insecurity and fear of abandonment, commonly linked to emotional volatility and lowered self-worth; these features are negatively associated with the self-esteem and emotional stability components of CSE (He et al., 2010; Li et al., 2019). Attachment avoidance involves emotional distancing to protect autonomy; although it may reduce immediate interpersonal strain, it is often associated with lower generalized self-efficacy and a more external locus of control, thereby undermining the self-efficacy and locus of control dimensions of CSE (Qu and Gan, 2012; Wei et al., 2011). By contrast, secure attachment, characterized by confidence in others’ availability and a stable sense of relational value, is consistently associated with higher self-esteem, greater self-efficacy, more effective emotion regulation, and superior resilience, yielding higher scores across CSE dimensions (Zhao and Wang, 2016).

Attachment orientations shape core self-evaluations by influencing self-appraisals and emotional coping. Meta-analytic evidence shows that insecure attachment, particularly attachment anxiety, is strongly associated with elevated self-criticism, thereby undermining core self-evaluative resources (Rogier et al., 2023). Related research further indicates that adverse family experiences erode core self-evaluation via reduced social support, contributing to maladaptive socioemotional outcomes (Su et al., 2024; Wang et al., 2021). Together, these findings underscore the role of attachment processes in preservice teachers’ personality development and career adaptability.

### 2.2 Attachment, academic emotions and core self-evaluations

Academic emotions are learners’ affective responses to tasks, outcomes, and contexts. Control-value theory locates these emotions in appraisals of perceived control and subjective value (Pekrun, 2006; Pekrun and Linnenbrink-Garcia, 2014). When learners appraise a task as important and attainable, they tend to experience positive states such as interest, enjoyment, hope, and pride, which foster intrinsic motivation, sustained attention, and deeper cognitive engagement (Linnenbrink, 2006; Mega et al., 2014; Pekrun et al., 2002). Conversely, appraisals of low control or low value elicit negative states such as anxiety, disappointment, and shame, which fragment cognitive resources, weaken motivation, and increase distress (Pekrun et al., 2002; Zhu and Zhang, 2017).

Recent empirical and methodological work further refines this picture: cross-cultural validation studies and reviews link interpersonal trust and attachment security to prosocial and moral emotions relevant for academic engagement (Oros et al., 2023; Martins et al., 2021); network-based psychometric analyses complement factor models and map the interactive structure of academic emotions in ways congenial to control-value assumptions (Mattsson et al., 2020); research on achievement goals shows that mastery and approach orientations selectively predict positive academic emotions such as enjoyment and hope (Martínez and Martins, 2025); and studies of psychological flexibility indicate it can mediate the effect of positive academic emotions on engagement and achievement, pointing to actionable mechanisms for intervention (López-Crespo et al., 2025).

Attachment orientations shape these appraisal processes and thereby influence core self-evaluations, a higher-order trait comprising self-esteem, generalized self-efficacy, emotional stability, and locus of control (Judge et al., 2003). Secure attachment is linked to more frequent positive academic emotions and to higher self-esteem and self-efficacy (Mikulincer and Shaver, 2019; Yang et al., 2013). Anxious and avoidant patterns tend to amplify negative academic emotions and to undermine emotional stability and perceived control (Mikulincer, 1998; Ma and Li, 2009). For preservice teachers, positive academic emotions bolster teaching confidence and classroom engagement, while persistent anxiety or shame promotes avoidance, increases burnout risk, and impairs emerging professional competence (Linnenbrink, 2006; Pekrun et al., 2002; Frenzel et al., 2009).

The present study therefore tests a mediation model in which attachment influences core self-evaluations via academic emotions, aiming to clarify how early relational experiences shape the affective and self-evaluative processes central to preservice teachers’ professional development (Judge et al., 2003; Pekrun, 2006).

### 2.3 The present study

Grounded in attachment theory and the control–value framework of academic emotions, this study surveyed 307 preservice teachers to examine how attachment orientations (anxiety, avoidance, closeness–dependence) predict core self-evaluations (CSE), with a focus on indirect effects via four academic emotions (disappointment, shame, interest, pride). By foregrounding the preservice phase as a formative period for CSE development, integrating attachment-based relational dispositions with control–value–informed emotional processes, and situating the analysis within a non-Western teacher education context, the study aims to clarify how early relational orientations are translated into foundational professional self-evaluations. Hierarchical regression first assessed the direct effects of attachment dimensions on CSE, followed by the inclusion of academic emotions to evaluate their incremental explanatory power. Bootstrapped mediation analyses then tested whether negative emotions (disappointment, shame) mediate the effect of attachment anxiety on CSE, and whether positive emotions (interest, pride) mediate the effect of closeness–dependence.

Based on this analytic sequence, we hypothesized that:

*H1*: Attachment anxiety and avoidance negatively predict CSE, whereas closeness–dependence positively predicts CSE.

*H2*: Negative academic emotions mediate the effect of attachment anxiety on CSE, and positive academic emotions mediate the effect of closeness–dependence on CSE.

*H3*: Among the mediators, disappointment is expected to exert the strongest negative effect and pride the strongest positive effect on CSE.

## 3 Methods

### 3.1 Participants and procedure

A multistage stratified convenience sample of full-time undergraduates in teacher education programs from four universities across North, East, South, and Southwest China, including centrally administered and key provincial normal universities, was recruited. Of 320 distributed questionnaires, 307 were valid, yielding a 95.9% valid response rate. The sample included 58 males (18.9%) and 249 females (81.1%), aged 18–24 years (*M* = 20.7, SD = 1.43), representing first- through fourth-year students.

Data were collected via standardized group administration. With approval from instructors and departmental administrators, surveys were administered in common courses (e.g., Education, Psychology) or professional classes. Trained examiners read a standardized script emphasizing voluntary participation, anonymity, confidentiality, and the right to withdraw. All participants provided written informed consent and completed questionnaires independently in quiet classrooms under supervision of at least two examiners. Each session lasted approximately 30 min. The study protocol was approved by the Wenzhou University Ethics Committee and conducted in accordance with ethical guidelines.

To ensure data quality, questionnaires with more than 5% missing data were excluded. Responses exhibiting patterned answering (same option on more than 10 consecutive Likert items) or logical inconsistencies identified via reverse-scored items were also removed. After screening, 307 questionnaires remained for analysis.

### 3.2 Measures

#### 3.2.1 Core self-evaluation scale

The Core Self-Evaluation Scale (CSES), originally developed by Judge et al. (1998) and later adapted into Chinese by Du et al. (2012), was employed to measure participants’ core self-evaluations. This unidimensional, self-report instrument contains 10 items rated on a 5-point Likert scale (1 = strongly disagree to 5 = strongly agree). Items 2, 3, 5, 7, 8, and 10 are reverse-scored. Higher total scores reflect higher levels of core self-evaluation. A sample item is: “Overall, I am satisfied with myself.” In the current study, the scale demonstrated good internal consistency, with a Cronbach’s alpha of 0.843.

#### 3.2.2 Revised adult attachment scale

The Revised Adult Attachment Scale (AAS; Collins and Read, 1990; Chinese version: Wu et al., 2004) is an 18-item, 5-point Likert scale measuring three dimensions: Closeness, Dependence, and Anxiety, with seven reverse-scored items (2, 7, 8, 13, 16, 17, 18). Closeness and Dependence can be combined into a single composite by summing item scores and dividing by 12. Sample items include “I find it difficult to fully trust others” (reverse-scored) and “I do not mind people getting close to me.” In this study, internal consistency was acceptable for the Closeness–Dependence composite (*α* = 0.720), and for the individual subscales: Closeness α = 0.612, Dependence α = 0.630, Anxiety α = 0.812.

#### 3.2.3 General academic emotions questionnaire for college students

Academic emotions were assessed using the General Academic Emotions Questionnaire (Ma, 2008), which evaluates 10 common emotions: interest, pleasure, pride, hope, relaxation, anger, anxiety, shame, disappointment, and boredom. The 88-item, 5-point Likert scale is organized into 10 subscales corresponding to each emotion and describes general learning-related scenarios. Sample items include: “I easily feel annoyed when studying,” “I feel happy while studying,” and “I am confident in my learning.” In the present study, Cronbach’s α for the 10 subscales ranged from 0.772 to 0.891, indicating acceptable to good internal consistency.

### 3.3 Data analysis

Data were analyzed using SPSS 21.0. First, as a preliminary step, stepwise multiple regression was employed to explore and screen predictors that showed independent associations with core self-evaluations. Second, guided by Pekrun’s (2006) theoretical framework on academic emotions, a hierarchical regression model was specified to examine the incremental contribution of distinct variable blocks. Finally, mediation analyses were conducted with Hayes’ PROCESS macro (version 3.3), using Model 4 and bootstrap resampling (5,000 samples) to estimate indirect effects. All tests were two-tailed and statistical significance was set at *p* < 0.05.

## 4 Results

### 4.1 Descriptive statistics and correlation analysis

Descriptive statistics indicated variable means between 2.6 and 3.9. Pearson correlations revealed that most associations among attachment dimensions, academic emotions, and core self-evaluation were significant at *p* < 0.001. Closeness and dependence were positively correlated with core self-evaluation and positive emotions (e.g., pride, pleasure) and negatively correlated with negative emotions (e.g., disappointment, anxiety). Attachment anxiety showed the opposite pattern, being negatively correlated with core self-evaluation and positively correlated with negative emotions. These results support the theoretical model and justify subsequent mediation analyses. Complete results are provided in Appendix Table 1.

### 4.2 Multiple regression results

We examined the predictive effects of adult attachment and academic emotions on core self-evaluations using regression analyses. The analysis proceeded in two stages: an exploratory stepwise regression to screen predictors, followed by hierarchical regression to test the theoretical contribution of variable blocks.

#### 4.2.1 Preliminary variable screening via stepwise regression

As an exploratory step, stepwise regression was used to identify potential predictors. Eight variables entered the final model: attachment anxiety and closeness–dependence, and the academic emotions disappointment, interest, pride, shame, hope, and pleasure. The model accounted for a substantial proportion of variance in core self-evaluations (*R*^2^ = 0.617). The incremental variance explained by each predictor was as follows: disappointment (45.0%), anxiety (6.7%), interest (5.6%), pride (1.4%), closeness–dependence (1.0%), shame (0.8%), hope (0.6%), and pleasure (0.6%); all ΔR^2^ values were statistically significant (*p*s < 0.05). See Appendix Table 2 for full results.

#### 4.2.2 Hierarchical regression analysis

Guided by Pekrun’s (2006) framework on academic emotion, variables were entered in three theoretical blocks. In Step 1 (attachment only), attachment anxiety (*β* = −0.404, *p* < 0.001) and closeness–dependence (*β* = 0.233, *p* < 0.001) both significantly predicted core self-evaluations. After adding negative academic emotions in Step 2, disappointment remained a strong negative predictor (*β* = −0.528, *p* < 0.001) while shame was nonsignificant; the effects of anxiety (*β* = −0.220, *p* < 0.001) and closeness–dependence (*β* = 0.152, *p* < 0.01) were attenuated but remained significant. When positive academic emotions were included in Step 3, closeness–dependence ceased to be significant (*β* = 0.105, *p* > 0.05), and the coefficient for pleasure unexpectedly reversed sign (*β* = −0.131, *p* < 0.05), suggesting multicollinearity. Full coefficients are reported in Appendix Table 3.

#### 4.2.3 Multicollinearity diagnostics and variable consolidation

Collinearity diagnostics identified severe multicollinearity between hope and pleasure (condition index > 30). The two variables theoretically belong to the same positive-activation emotion category (Pekrun, 2006). Principal component analysis extracted a single component (eigenvalue = 1.711) that explained 85.56% of the variance, with loadings of 0.925 for both items, supporting their combination. We therefore created a composite variable, labeled the hope–pleasure composite, to address collinearity. After substituting the composite into the model, its regression coefficient was positive and aligned with theoretical expectations, and the remainder of the model remained stable (see Appendix Table 4).

Overall, the analyses identified attachment anxiety, disappointment, shame, interest, pride, and the hope–pleasure composite as significant predictors of core self-evaluations, providing an empirical basis for subsequent mediation tests of academic emotions in the attachment → core self-evaluation pathway.

### 4.3 Mediation analysis of attachment and core self-evaluation among normal university students

Controlling for gender, the mediation effect of negative academic emotions (disappointment and shame) on the relationship between attachment anxiety and core self-evaluation (CSE) was examined. Results indicated that attachment anxiety significantly negatively predicted CSE. Even after including the two mediators, the effect of attachment anxiety on CSE remained significant, demonstrating that negative emotions (disappointment and shame) mediate the relationship between attachment anxiety and CSE. Specifically, attachment anxiety positively and significantly predicted shame, but shame’s predictive effect on CSE was not significant. Similarly, attachment anxiety significantly positively predicted disappointment, and disappointment significantly negatively predicted CSE. Detailed results are shown in Table 1.

**Table 1:** Mediation analysis of negative academic emotions (Disappointment and shame) in the relationship between attachment anxiety and core self-evaluations among normal university students.

Variable	CSE			Shame			Disappointment		
	*β*	*se*	*t*	*β*	*se*	*t*	*β*	*se*	*t*
Constant	5.343	0.139	38.586^***^	1.879	0.184	10.219^***^	1.815	0.115	15.736^***^
Gender	0.047	0.058	0.816	−0.024	0.089	−0.266	−0.018	0.075	−0.234
Attachment anxiety	−0.204	0.033	−6.221^***^	0.172	0.049	3.471^***^	0.307	0.038	8.105^***^
Disappointment	−0.549	0.047	−11.804^***^	0.395	0.068	5.825^***^			
Shame	0.003	0.037	0.081						
*R^2^*	0.518			0.205			0.182		
*F*	81.009^***^			26.036^***^			33.704^***^		

CSE = core self-evaluation, *p < 0.05, **p < 0.01, ***p < 0.001.

Mediation analyses using bootstrap resampling with 5,000 draws indicated that anxiety had a significant total effect on core self-evaluations (*β* = −0.371, 95% CI [−0.435, −0.303]). The direct effect was also significant (*β* = −0.204, 95% CI [−0.273, −0.134]), accounting for 54.9% of the total effect. The total indirect effect was significant (*β* = −0.168, 95% CI [−0.221, −0.120]) and accounted for 45.1% of the total effect, indicating a significant mediating role of academic emotions.

**Figure 1:**
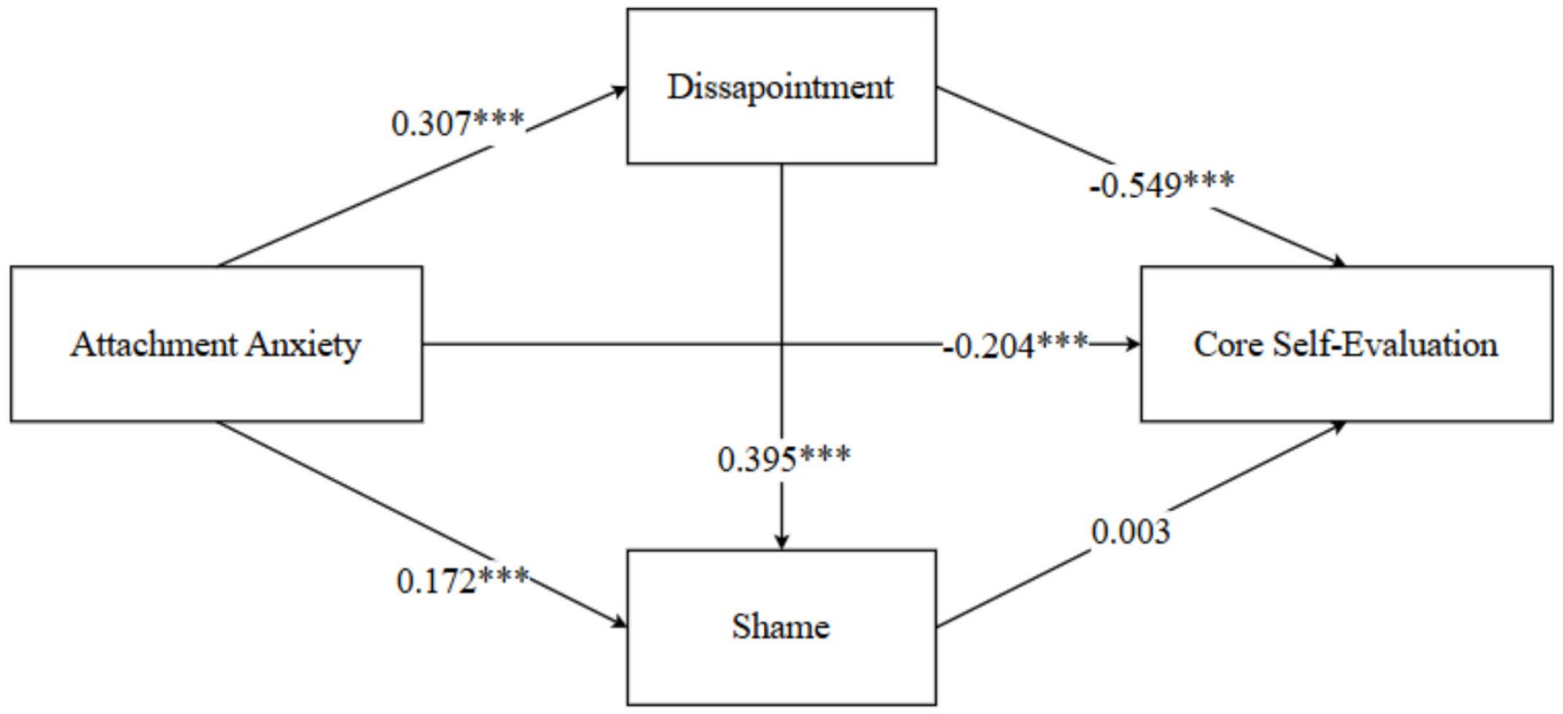
The mediating roles of disappointment and shame in the relationship between attachment anxiety and core self-evaluation. Path coefficients are standardized *β* values; ***p < 0.001.

Path-specific analyses further showed that only the indirect path from anxiety to core self-evaluations via disappointment was significant (*β* = −0.168, 95% CI [−0.221, −0.121]), explaining 45.4% of the total effect. In contrast, the indirect paths through shame alone and through the sequential pathway of disappointment and shame were not significant, as their 95% confidence intervals included zero. These findings indicate that disappointment serves as a significant negative partial mediator between anxiety and core self-evaluations, whereas the mediating role of shame and the proposed chain mediation were not supported. See Figure 1.

Controlling for gender, the mediating effects of positive academic emotions (interest and pride) in the relationship between attachment (closeness-dependence) and core self-evaluations among normal university students were examined. The results showed that closeness-dependence significantly and positively predicted core self-evaluations. Even after including the two mediating variables, the influence of closeness-dependence on core self-evaluations remained significant, indicating that positive academic emotions (interest and pride) mediate the relationship between closeness-dependence and core self-evaluations. The positive predictive effect of closeness-dependence on interest was not significant, while interest significantly predicted core self-evaluations. Similarly, closeness-dependence significantly positively predicted pride, which in turn significantly positively predicted core self-evaluations. See Table 2 for details. Hypotheses 1 and 2 were supported.

**Table 2:** Mediation effect analysis of positive academic emotions between closeness-dependence and core self-evaluations.

Variable	CSE			Interest			Pride		
	*β*	*se*	*t*	*β*	*se*	*t*	*β*	*se*	*t*
Constant	0.407	0.223	1.826^*^	0.281	0.236	1.189	2.717	0.178	15.282^***^
Gender	0.031	0.066	0.470	0.086	0.070	1.228	0.075	0.070	1.076
CD	0.369	0.051	7.301^***^	0.021	0.054	0.398	0.184	0.053	3.492^***^
Pride	0.332	0.069	4.844^***^	0.783	0.057	13.661^***^			
Interest	0.180	0.054	3.331^***^						
*R^2^*	0.373			0.401			0.045		
*F*	44.934^***^			67.509^***^			7.214^***^		

CSE = core self-evaluation; CD=Closeness–dependence. *p < 0.05, **p < 0.01, ***p < 0.001.

Mediation analyses based on the bootstrap method with 5,000 resamples showed that closeness–dependence had a significant total effect on core self-evaluations (*β* = 0.460, 95% CI [0.349, 0.567]). The direct effect was significant (*β* = 0.369, 95% CI [0.275, 0.471]), accounting for 80.2% of the total effect. The total indirect effect was also significant (*β* = 0.091, 95% CI [0.030, 0.152]), accounting for 19.8% of the total effect, indicating that positive academic emotions played a significant mediating role between closeness–dependence and core self-evaluations.

**Figure 2:**
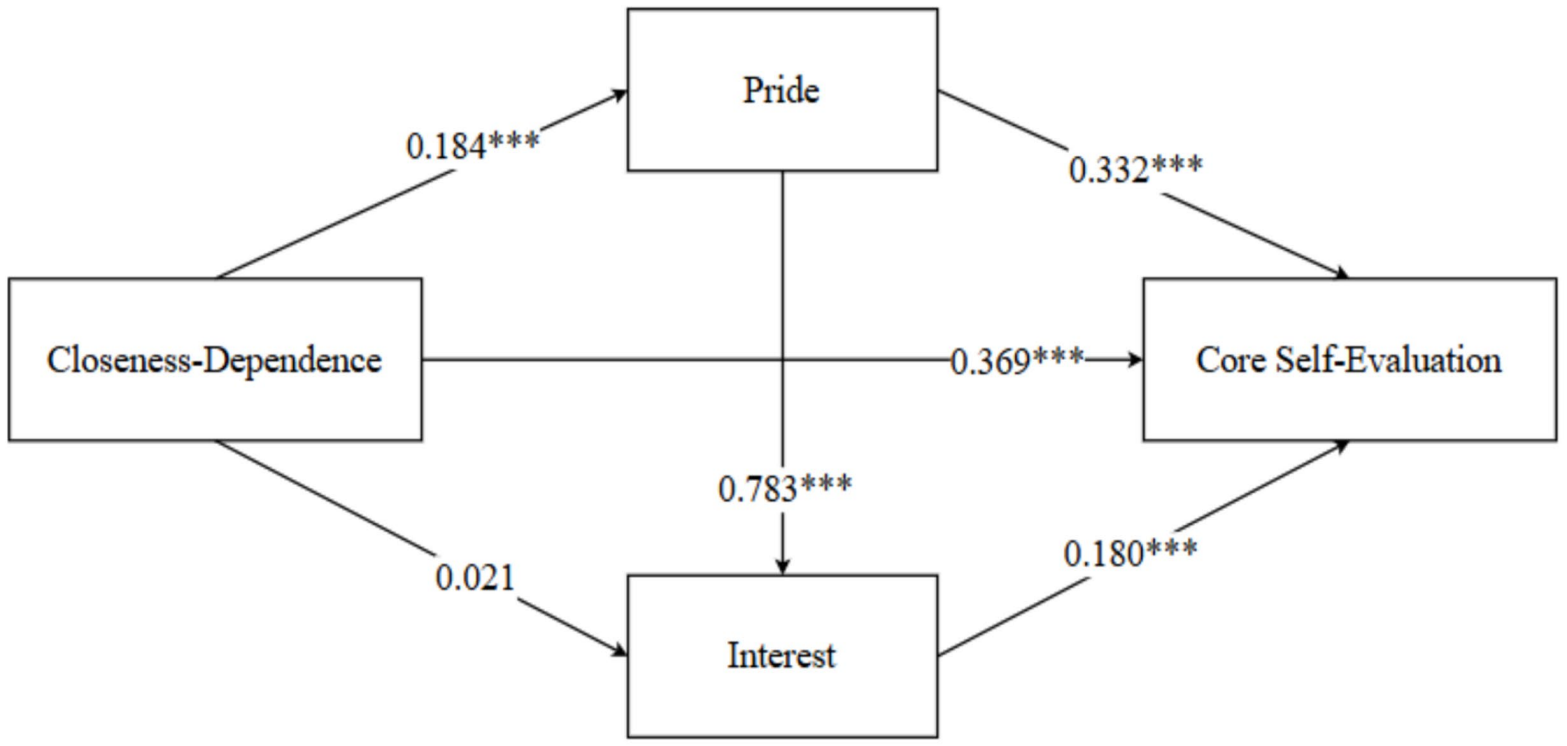
The mediating roles of pride and interest in the relationship between closeness-dependence and core self-evaluation. Note: Path coefficients are standardized *β* values; ***p < 0.001.

Further path-specific analyses revealed that the indirect effect via pride was significant (closeness–dependence → pride → core self-evaluations; β = 0.061, 95% CI [0.020, 0.110]), accounting for 19.8% of the total effect. In contrast, the indirect effect via interest alone was not significant (β = 0.004, 95% CI [−0.017, 0.030]). The chain mediation pathway from closeness–dependence to core self-evaluations via pride and interest was significant (β = 0.026, 95% CI [0.006, 0.051]), accounting for 5.6% of the total effect. These results indicate that pride serves as a significant independent mediator between closeness–dependence and core self-evaluations and also exerts a chain mediating effect by enhancing interest, whereas the independent mediating role of interest was not supported. See Figure 2. Hypothesis 3 was fully supported.

## 5 Discussion

### 5.1 Discussion of key predictors in the regression analysis

Regression results indicated that among negative academic emotions, disappointment was the strongest predictor of core self-evaluations (CSE), accounting for a significant proportion of variance, consistent with recent findings that negative academic emotions are inversely related to self-efficacy and academic adjustment outcomes; for example, academic self-efficacy has been shown to mediate the influence of negative academic emotions on maladaptive academic behaviors such as procrastination, underscoring how adverse emotional experiences undermine students’ confidence and engagement in academic contexts (Chen et al., 2024). This aligns with recent person-centered evidence showing that students with high anxiety and disengagement report lower CSE, highlighting the central role of negative academic emotions in academic adaptation (Karagiannopoulou and Ntritsos, 2025).

Attachment anxiety explained 18.3% of the variance in core self-evaluations (CSE), reflecting that chronic relational insecurity undermines self-worth and self-competence; consistent with recent research showing that insecure attachment is negatively associated with self-esteem and related self-evaluative constructs, which in turn predict lower core self-evaluations and broader psychological adjustment outcomes (Song et al., 2024). This is consistent with cross-cultural findings that insecure attachment reduces psychological capital and academic engagement (Paloș et al., 2023).

Attachment closeness–dependence contributed 15.7% of variance, underscoring secure, supportive relationships in enhancing self-efficacy and positive emotions like interest and pride (Wei et al., 2011). Similarly, parental and romantic attachment studies show secure bonds enhance CSE via belonging and emotional security (Zhang et al., 2024; Song et al., 2024).

When positive emotions (interest, pride) were added, shame’s unique contribution rose to 11.4% (*p* < 0.01), suggesting that self-reproach interacts with positive affect rather than being offset by it (Leach, 2017). Recent work highlights that mixed emotional states in demanding contexts affect self-concept beyond single-emotion models (Tao et al., 2025).

### 5.2 Analysis of mediating effects

Our analyses indicate that academic negative emotion, specifically disappointment, functions as a significant mediator of the relationship between attachment anxiety and CSE. Consistent with contemporary research on attachment and emotional processes, insecure attachment (including attachment anxiety) has been linked to greater difficulties in emotion regulation, which in turn negatively influence self-related beliefs, competence appraisals, and wellbeing (Erkoreka et al., 2021). Specifically, individuals high in attachment anxiety may transfer relational insecurity into broader evaluative contexts, exacerbating negative emotional experiences and undermining perceived competence and efficacy in achievement-related domains, thus accounting for their lower core self-evaluations. This pathway dovetails with empirical work showing that negative academic emotions transmit dispositional vulnerabilities into maladaptive academic outcomes such as disengagement and burnout (Zhang et al., 2024), suggesting that interventions targeting the reduction of disappointment in evaluative situations may help buffer the adverse impact of attachment anxiety on CSE.

By contrast, academic positive emotions—notably interest and pride—partially mediated the association between attachment closeness–dependence and CSE. Secure attachment relations foster autonomy and competence experiences that act as antecedents to sustained interest and episodic pride; these positive affective states, in turn, reinforce mastery beliefs and task persistence, thereby supporting higher CSE (Park et al., 2022). This pattern is in line with recent findings that learner profiles characterized by high engagement and intrinsic motivation show clustered positive academic emotions that bolster self-efficacy and adaptive adjustment (Karagiannopoulou and Ntritsos, 2025). From a resource perspective, positive emotions function as activating mechanisms that broaden individuals’ cognitive and motivational resources, supporting sustained engagement and academic outcomes; recent empirical work within the broaden-and-build framework has found that positive emotional experiences are positively associated with increased psychological resources, life satisfaction, and academic engagement, indicating that positive affect contributes to the accumulation of personal and cognitive resources that facilitate adaptive functioning and performance (Wang et al., 2025).

When positive and negative emotions were modeled simultaneously, results pointed to a more nuanced, compound emotional mediation, with shame emerging as a salient mediator; this aligns with recent evidence that difficulties in emotion regulation mediate the effects of shame-proneness on adverse outcomes, underscoring that the mediating role of affect depends on specific emotional configurations rather than isolated emotions alone (Erbildim and Nweke, 2025). This finding accords with contemporary teacher- and learner-emotion research emphasizing the dynamic, co-occurring nature of emotional states and their stronger influence on self-perception and performance than single-emotion models (Tao et al., 2025). Practically, these results imply that interventions should both cultivate positive academic emotions (e.g., interest, pride) and include strategies for regulating complex negative self-conscious emotions such as shame, because fostering positive affect and concurrently attenuating maladaptive self-evaluative emotions may produce the most robust gains in CSE.

### 5.3 Educational practices: strategies and recommendations

A dual-mentor model pairing academic advisors with emotional mentors, such as trained counselors or experienced peers, is recommended to address attachment anxiety and negative academic emotions (Levy and Blatt, 2003; Mikulincer and Shaver, 2019). Given that the present study found attachment anxiety to explain 18.3% of variance in core self-evaluations (CSE), the dual-mentor model should prioritize sustained relational support and targeted relationship-repair interventions for preservice teachers who exhibit high attachment anxiety.

Embedding an Academic Emotion Awareness and Management module that draws on Gross’s process model and Pekrun’s control–value theory and that uses video reflection, cognitive reappraisal, and peer-led strength sessions can enhance interest and pride while reducing disappointment and shame (Pekrun, 2006; Cohen et al., 2023; Frenzel et al., 2009). Because this study identified disappointment as the strongest single predictor of CSE among negative academic emotions, the module should include explicit strategies to prevent and mitigate disappointment, such as transparent assessment criteria, increased formative assessment, and structured, constructive feedback that emphasizes incremental improvement rather than summative judgment. Strengthening emotional and motivational resources has been shown to improve engagement and academic outcomes via CSE (Xu, 2024; Paloș et al., 2023). Interventions should also deliberately cultivate interest and pride, since interest and pride partially mediated the relation between attachment closeness–dependence and CSE in our data; instructional design therefore ought to offer autonomy-supportive choices, tiered mastery tasks, and visible opportunities for attributable success that foster authentic pride.

Practical training should integrate scaffolded mastery experiences in microteaching labs, structured reflective-practice debriefs informed by recent research on reflective scaffolding, and systematic, immediate feedback aligned with Hattie and Timperley’s (2007) model to incrementally strengthen self-efficacy and professional pride. Empirical evidence shows that structured reflection prompts and repeated experience–reflection cycles enhance metacognitive reasoning and adaptive instructional decisions (Anselmann, 2023), while microteaching with multi-source, timely feedback improves reflective awareness, confidence, and skill acquisition (Arsal, 2014; Kelleci Alkan et al., 2024). Organizing microteaching into short, achievable cycles fosters effort-based successes that reinforce competence and reduce negative affect. To sustain these gains, longitudinal support systems incorporating emotion-regulation portfolios, mentor logs, and alumni follow-ups are needed, as attachment-informed research indicates that secure relational support predicts stronger and more stable professional self-evaluations through enhanced teacher–student relationships and self-efficacy (Nazaralian and Aghaei, 2025). Such sustained relational scaffolding is particularly critical for individuals high in attachment anxiety, whose professional confidence is more vulnerable without ongoing supportive relationships.

Attachment and academic emotions operate within cultural contexts. In Chinese educational settings, strong teacher–student relations tend to promote positive academic emotions and motivation, while hierarchical relations and high-stakes assessment may intensify evaluative emotions such as disappointment and shame (Wang et al., 2024). A collectivistic school climate has been associated with greater engagement and emotional competence and can amplify the social meaning of feedback and peer comparison (Liu and Wang, 2023). Research in Chinese classroom contexts further shows close links among academic emotions, engagement, and self-efficacy, and indicates that school climate influences achievement through affective pathways (Hu et al., 2024; Li et al., 2017). Teacher–student relationships have also been linked to social–emotional competence via empathy and self-efficacy, highlighting culturally salient mechanisms that shape emotional and motivational outcomes (Yu, 2023). Accordingly, culturally attuned implementation is essential: mentors should be trained in culturally competent feedback, peer-mentor groups should reflect existing cohort structures to leverage collective motivation, and emotion-management skills should be framed as instrumental to academic success rather than remedial.

To maximize uptake and effectiveness, interventions should be culturally attuned: train mentors in culturally competent feedback, design peer-mentor groups that mirror existing cohorts to harness collective motivation, present emotion-management skills as instrumental to academic success rather than remedial, and use anonymized portfolios or class-level reflection to protect face while reducing shame. Monitoring should combine quantitative CSE indices with qualitative indicators of face-related distress and perceived teacher support to detect culturally specific risk and resilience pathways. In evaluation frameworks, “disappointment” should be treated as a primary outcome to monitor, and interest, pride, and attachment security should be included as intermediate process measures to assess whether the intervention acts on the hypothesized mechanisms. In sum, two priority, actionable strategies emerge from the present findings: first, reduce academic disappointment through assessment reform and feedback practices; second, increase opportunities for attainable mastery that build interest and pride and thereby repair the self-evaluative deficits associated with attachment anxiety.

### 5.4 Limitations and directions for future research

This study has several limitations. The cross-sectional design precludes causal inference, and reliance on self-report measures may introduce common method bias. The model also focuses on individual-level factors while neglecting contextual influences such as classroom climate and institutional culture. In addition, the attachment measure shows psychometric weaknesses: although the combined closeness–dependence scale was acceptable (*α* = 0.720), the subscales demonstrated low reliability (closeness α = 0.612; dependence α = 0.630), likely attenuating effect sizes. Methodologically, the study remains purely quantitative. As the reviewer noted, without complementary qualitative data, the analysis offers limited insight into the underlying psychological processes behind the observed associations. Future work should use longitudinal or experimental designs, improved measurement tools, and multi-informant or multilevel approaches. Incorporating qualitative methods—such as interviews or focus groups—would also provide deeper contextual understanding and strengthen the interpretive validity of the findings.

## 6 Conclusion

This study shows that preservice teachers’ attachment orientations shape their core self-evaluations (CSE) through specific academic emotions. Disappointment and shame mediate the impact of attachment anxiety, whereas pride and interest mediate the benefits of attachment security. These results extend attachment theory into teacher education and highlight emotional processes, both negative and positive, as central to the formation of professional self-concept.

Practically, teacher preparation should prioritize relational and emotional supports, such as dual-mentor systems, reflective practice, emotion-regulation training, and scaffolded mastery experiences with structured feedback to strengthen CSE and resilience. Future research should assess the long-term effectiveness of these strategies and examine contextual moderators, including school climate and mentoring quality, to inform scalable interventions for cultivating emotionally competent and self-assured educators.

## Data Availability

The raw data supporting the conclusions of this article will be made available by the authors, without undue reservation.
