# Supplementary material for: Understanding the role of preservice teachers’ attachment in shaping core self-evaluations: the mediating effect of academic emotions in teacher education contexts
Source: Front Psychol. 2026 Jan 29;17:1717612. doi: 10.3389/fpsyg.2026.1717612 (PMC12895784; doi:10.3389/fpsyg.2026.1717612)
Supplement: Supplementary file 1 [file Table_1.DOCX]

**Table 1.** Descriptive Statistics and Correlation Analysis of the Main Variables

| Variable | 1 | 2 | 3 | 4 | 5 | 6 | 7 | 8 | 9 | 10 | 11 | 12 | 13 |
| --- | --- | --- | --- | --- | --- | --- | --- | --- | --- | --- | --- | --- | --- |
| 1 | 1 |  |  |  |  |  |  |  |  |  |  |  |  |
| 2 | -0.46^**^ | 1 |  |  |  |  |  |  |  |  |  |  |  |
| 3 | 0.42^**^ | 0.13^*^ | 1 |  |  |  |  |  |  |  |  |  |  |
| 4 | -0.20^**^ | 0.63^**^ | 0.30^**^ | 1 |  |  |  |  |  |  |  |  |  |
| 5 | -0.37^**^ | 0.68^**^ | 0.19^**^ | 0.71^**^ | 1 |  |  |  |  |  |  |  |  |
| 6 | -0.33^**^ | 0.63^**^ | 0.08 | 0.67^**^ | 0.58^**^ | 1 |  |  |  |  |  |  |  |
| 7 | 0.37^**^ | 0.12^*^ | 0.63^**^ | 0.25^**^ | 0.09 | 0.08 | 1 |  |  |  |  |  |  |
| 8 | 0.75^**^ | -0.24^**^ | 0.64^**^ | 0.03 | -0.13^*^ | -0.19^**^ | 0.52^**^ | 1 |  |  |  |  |  |
| 9 | 0.66^**^ | -0.54^**^ | 0.12^*^ | -0.52^**^ | -0.56^**^ | -0.58^**^ | 0.22^**^ | 0.53^**^ | 1 |  |  |  |  |
| 10 | -0.52^**^ | 0.65^**^ | -0.20^**^ | 0.44^**^ | 0.47^**^ | 0.62 | -0.1 | -0.49^**^ | -0.60^**^ | 1 |  |  |  |
| 11 | -0.67^**^ | 0.48^**^ | -0.32^**^ | 0.26^**^ | 0.47^**^ | 0.43^**^ | -0.27^**^ | -0.55^**^ | -0.53^**^ | 0.54^**^ | 1 |  |  |
| 12 | -0.33^**^ | 0.20^**^ | -0.20^**^ | 0.18^**^ | 0.31^**^ | 0.15^**^ | -0.20^**^ | -0.32^**^ | 0.22^**^ | 0.27^**^ | 0.43^**^ | 1 |  |
| 13 | 0.43^**^ | -0.1 | 0.34^**^ | 0 | -0.22^**^ | -0.09 | 0.29^**^ | 0.41^**^ | -0.30^**^ | -2.45^**^ | -0.52^**^ | -0.49^**^ | 1 |
| *M±SD* | *2.69±0.56* | *3.35±0.49* | *3.43±0.68* | *3.55±0.46* | *3.84±0.47* | *2.99±0.61* | *3.01±0.64* | *3.19±0.63* | *2.69±0.60* | *3.03±0.51* | *3.30±0.56* | *3.35±0.52* | *2.87±0.78* |

*Note: The variable names 1–13 represent the following constructs:*

*disappointment,pride, shame, pleasure, hope, interest, anger, anxiety, boredom, relaxation, core self-evaluation(****CSE), closeness–dependence and*** *anxiety (attachment), respectively.*

**p < 0.05, **p < 0.01.*

**Table 2 Summary of Stepwise Multiple Regression Analysis of Attachment and Academic Emotions on Core Self-Evaluation**

| Step | *R* | *R²* | *ΔR²* | *F* | *ΔF* | *B* | *β* |
| --- | --- | --- | --- | --- | --- | --- | --- |
| Intercept |  |  |  |  |  | 3.250 |  |
| 1 | .671 | .450 | .450 | 249.393^***^ | 249.393^***^ | -0.322 | -0.323 |
| 2 | .719 | .517 | .067 | 162.394^***^ | 41.929^***^ | -0.158 | -0.219 |
| 3 | .757 | .573 | .056 | 135.284^***^ | 39.709^***^ | 0.172 | 0.187 |
| 4 | .766 | .586 | .014 | 107.066^***^ | 10.152^**^ | 0.183 | 0.158 |
| 5 | .772 | .597 | .010 | 89.066^***^ | 7.644^**^ | 0.114 | 0.105 |
| 6 | .778 | .605 | .008 | 76.597^***^ | 6.344^*^ | -0.101 | -0.121 |
| 7 | .782 | .611 | .006 | 67.020^***^ | 4.381^*^ | 0.203 | 0.169 |
| 8 | .785 | .617 | .006 | 59.940^***^ | 4.650^*^ | -0.161 | -0.131 |

*Note: The variable names 1–8 represent the following constructs: disappointment, anxiety (attachment), interest, pride, closeness-dependence (attachment), shame, hope, and pleasure, respectively. *p < 0.05, **p < 0.01, ***p < 0.001.*

**Table 3** Hierarchical Regression Analysis Results

| Step | PVWS | Step 1 |  | Step 2 |  | Step 3 |  |
| --- | --- | --- | --- | --- | --- | --- | --- |
|  |  | *β* | *t* | *β* | *t* | *β* | *t* |
| AA | Attachment Anxiety | -.404 | -7.390^***^ | -.220 | -4.538^***^ | -.219 | -4.816^***^ |
|  | CD | .233 | 4.267^***^ | .152 | 3.334^**^ | .105 | 2.449 |
| ANE | Disappointment |  |  | -.528 | -11.418^***^ | -.323 | -6.378^***^ |
|  | Shame |  |  | .002 | .054 | -.121 | -2.641^**^ |
| APE | Interest |  |  |  |  | .187 | 3.558^***^ |
|  | Pride |  |  |  |  | .158 | 2.763^**^ |
|  | Hope |  |  |  |  | .169 | 2.813^**^ |
|  | Pleasure |  |  |  |  | -.131 | -2.156^*^ |
| RMS | F | 68.580 |  | 86.413 |  | 59.940 |  |
|  | R² | .311 |  | .534 |  | .617 |  |
|  | ΔF | 68.580^***^ |  | 72.147^***^ |  | 16.139^***^ |  |
|  | ΔR² | .311 |  | .223 |  | .083 |  |

***Note:AA = Adult Attachment, CD=Closeness–dependence, ANE = Academic Negative Emotions, APE = Academic Positive Emotions, RMS = Regression Model Summary,***

***PVWS = Predictor Variables Within Step,*** **p < 0.05, **p < 0.01, ***p < 0.001.*

**Table 4**Regression Analysis Results After Combining Variables Using Principal Component Analysis

| Step | PVWS | Step 1 | | Step 2 | | Step 3 | |
| --- | --- | --- | --- | --- | --- | --- | --- |
|  |  | *β* | *t* | *β* | *t* | *β* | *t* |
| AA | Attachment Anxiety | -.404 | -7.390^***^ | -.220 | -4.538^***^ | -.245 | -5.420^***^ |
|  | CD | .233 | 4.267^***^ | .152 | 3.334^**^ | .108 | 2.483^*^ |
| ANE | Disappointment |  |  | -.528 | -11.418^***^ | -.340 | -6.684^***^ |
|  | Shame |  |  | .002 | .054 | -.120 | -2.578^**^ |
| APE | Interest |  |  |  |  | .173 | 2.997^**^ |
|  | Pride |  |  |  |  | .154 | 2.963^**^ |
|  | Hope- pleasure |  |  |  |  | .037 | .611 |
| RMS | F | 68.580^***^ |  | 86.413^***^ |  | 65.571^***^ |  |
|  | R² | .311 |  | .534 |  | .606 |  |
|  | ΔF | 68.580^***^ |  | 72.147^***^ |  | 18.150^***^ |  |
|  | ΔR² | .311 |  | .223 |  | .072 |  |

***Note:***

***PVWS = Predictor Variables Within Step***

***AA = Adult Attachment***

***CD=Closeness–Dependence***

***ANE = Academic Negative Emotions***

***APE = Academic Positive Emotions***

***RMS = Regression Model Summary***

**p < 0.05, **p < 0.01, ***p < 0.001.*
